# Wernicke’s Encephalopathy Following Pregnancy Loss: A Case Report and Literature Review

**DOI:** 10.1155/crnm/4526948

**Published:** 2026-08-20

**Authors:** Himel Kumar Biswas, Monisha Basak, Mosarrat Mahjabeen, Nazia Ahmed, Rama Biswas

**Affiliations:** ^1^ Department of Neurology, Square Hospitals Ltd., Dhaka, Bangladesh; ^2^ Department of Internal Medicine, Square Hospitals Ltd., Dhaka, Bangladesh; ^3^ Department of Accident and Emergency, Square Hospitals Ltd., Dhaka, Bangladesh

## Abstract

A 40‐year‐old female with no history of alcohol use presented with altered mental status and generalized weakness ten days after dilation and curettage for missed abortion, complicated by persistent vomiting and electrolyte imbalances. Examination revealed confusion and disorientation, without nystagmus, ophthalmoplegia, or ataxia. Brain MRI showed characteristic symmetrical periventricular signal abnormalities consistent with Wernicke’s encephalopathy. She was treated with intravenous thiamine (500 mg three times daily for five days), followed by oral supplementation, with gradual resolution of confusion. She was discharged on the 13th hospital day in a hemodynamically and neurologically stable condition. This case highlights that nonalcoholic Wernicke’s encephalopathy can occur following surgical procedures complicated by prolonged vomiting and nutritional deficits. Early recognition and prompt thiamine replacement are essential for favorable outcomes.

## 1. Introduction

Wernicke’s encephalopathy (WE) is an acute neurological disorder caused by thiamine (vitamin B1) deficiency and represents a medical emergency [1]. It is classically associated with chronic alcohol use; however, nonalcoholic etiologies such as malnutrition, prolonged vomiting, postsurgical states, and pregnancy‐related complications are increasingly recognized [2]. The classical clinical triad of WE consists of confusion, ophthalmoplegia, and ataxia [3]. Nevertheless, fewer than one‐third of patients present with all three features, often resulting in delayed or missed diagnosis, particularly after obstetric complications associated with prolonged nutritional deprivation, nonalcoholic pregnancy‐related complications [4]. Early recognition and prompt administration of intravenous thiamine are crucial to prevent irreversible neurological damage or progression to Korsakoff syndrome [5]. Here, we report a case of nonalcoholic WE in a 40‐year‐old woman following obstetric complications, presenting with isolated confusion without ocular or cerebellar signs.

## 2. Case Presentation

A 40‐year‐old female, was admitted to our hospital with altered mental status and generalized weakness. She had no history of alcohol use or chronic liver disease. She has a history missed abortion managed 1 year earlier. Recently, she underwent dilation and curettage (D&C) for another missed abortion 10 days prior to admission. The perioperative period was complicated by persistent vomiting, poor oral intake, and electrolyte imbalance.

Upon admission, she was conscious but confused. She was disoriented with impaired attention and altered sensorium. Her Glasgow Coma Scale (GCS) was E_3_V_3_M_5_. There were no ocular abnormalities; specifically, no nystagmus, ophthalmoplegia, or pupillary abnormalities were noted. Cerebellar examination revealed no ataxia, and there were no focal neurological deficits on the clinical examination. Laboratory investigations (Table 1) demonstrated moderate anemia, elevated inflammatory markers, hypoalbuminemia, hypokalemia, mild hyponatremia, hypomagnesemia, and hypocalcemia, consistent with nutritional deficiency and metabolic imbalance.

**TABLE 1:** Laboratory investigations.

T/N; day	Normal range	19/10	20/10	21/10	22/10	23/10	24/11	25/10	26/10	27/10	28/10	31/10	01/11
Hb	12–15 gm/dL	7.40		9.30				8.90			10.1	10.70	10.1
WBC	4–10.0 K/μL	5.63		6.24				3.96			6.71	5.62	5.66
PLT	150–410 K/μL	238		178				190			278	333	307
CRP	< 10 mg/L	8.8			41.4			31.7					< 5
S. Folate	3‐4 ng/mL						3.8						
Vitamin B12	197–771 pg/mL						804.1						
ALT	< 35 U/L			133				98					
AST	14–36 U/L			43									
Bilirubin	0.2–1.2 mg/dL			1.0									
Vitamin D	30–100 ng/mL						< 8						
Na	135–145 mmol/L		135	135	134	138	138	139	136	139	134	133	134
K	3.5–5 mmol/L		3	3.2	2.9	3	2.5	3.3	3	3.5	3.8	4.9	4.4
Creatinine	0.5–1.04 mg/dL				0.5								
Albumin	3.5–5.0 g/dL						2.4					3.8	
Ca	8.5–10.5 mg/dL				8.1								
Mg	1.6–2.3 mg/dL				1.4		1.9						
Phosphorus	2.5–4.5 mg/dL				2.9								
Ferritin	Female < 50 years: 6.24–137 Ng/mL			253									
Iron	Female 37–170 Mcg/mL			76									
TIBC	Female 265–497 Mcg/mL			304									

**FIGURE 1:**
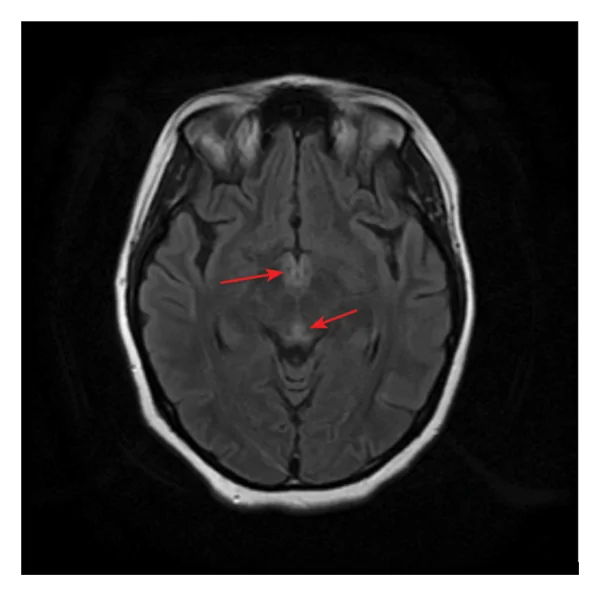
Symmetrical T2/FLAIR hyperintensities involving the bilateral medial thalami, periaqueductal gray matter, and mammillary bodies are demonstrated.

Magnetic resonance imaging (MRI) of the brain revealed characteristic symmetrical signal abnormalities in periventricular regions (Figure 1) consistent with WE.

Based on the clinical presentation, nutritional risk factors, and MRI findings, a diagnosis of nonalcoholic WE was established. The patient was treated promptly with intravenous thiamine (500 mg tid for 5 days) later transitioned to oral supplementation upon clinical improvement. Her confusion gradually resolved, and she was discharged on the 13th day of hospitalization in a hemodynamically and neurologically stable condition.

## 3. Case Discussion

This case illustrates a nonalcoholic presentation of WE precipitated by miscarriage‐associated anorexia and metabolic stress. The diagnosis was supported by prolonged nutritional deprivation, biochemical evidence of starvation with electrolyte imbalance, and characteristic MRI findings. These features are clinically important, as they demonstrate that WE can occur in the absence of alcohol use and without the complete classical triad.

Although WE is traditionally linked to chronic alcoholism, increasing evidence shows that nonalcoholic causes are frequently underrecognized [6]. In such patients, the clinical presentation is often incomplete or atypical. Symptoms may be nonspecific, including generalized weakness, altered mental status, or autonomic instability, which can delay diagnosis. In the present case, prolonged reduced oral intake following miscarriage, along with ketonuria, hypoalbuminemia, and electrolyte disturbances, reflected a hypercatabolic and malnourished state. This metabolic stress likely led to rapid depletion of thiamine stores. Previous studies have shown that thiamine deficiency can develop within weeks during periods of increased metabolic demand, even in individuals without prior nutritional risk [2].

Neuroimaging played a central role in establishing the diagnosis. The symmetric T2/FLAIR hyperintensities involving the medial thalami, periaqueductal gray matter, and mammillary bodies are well‐described radiological features of WE [7]. MRI is particularly valuable in nonalcoholic cases, where clinical suspicion is often low and neurological signs may be subtle or incomplete [2]. In this patient, the absence of diffusion restriction or contrast enhancement suggests an early and potentially reversible stage of the disease.

A comparison with the existing literature highlights pregnancy‐related conditions as important nonalcoholic risk factors for WE. These include hyperemesis gravidarum and postpregnancy nutritional deficiency [8]. Most reported cases focus on prolonged vomiting during pregnancy. In contrast, this case emphasizes miscarriage‐associated anorexia and psychological stress as potential precipitating factors. It demonstrates that significant nutritional deprivation after miscarriage, even without persistent vomiting, may be sufficient to trigger WE.

From a pathophysiological perspective, thiamine deficiency disrupts cerebral glucose metabolism, leading to mitochondrial dysfunction, oxidative stress, and neuronal injury in metabolically vulnerable brain regions [9]. The presence of hypokalemia and hypomagnesemia in this patient is clinically relevant. Magnesium is an essential cofactor for thiamine‐dependent enzymes, and its deficiency may impair intracellular thiamine utilization. Concurrent infection and systemic illness may further increase metabolic demand, thereby accelerating neurological injury.

Current international guidelines recommend prompt administration of high‐dose intravenous thiamine (500 mg three times daily) in patients with suspected WE, as delayed or inadequate treatment may lead to irreversible neurological injury, including persistent cognitive impairment and progression to Korsakoff syndrome [10]. Thiamine should be administered before or concomitantly with glucose‐containing fluids whenever possible to avoid exacerbating thiamine deficiency. In our patient, early recognition based on the clinical presentation and characteristic MRI findings, followed by prompt initiation of high‐dose intravenous thiamine, resulted in complete neurological recovery, underscoring the importance of timely diagnosis and treatment even in atypical, nonalcoholic presentations.

Table 2 summarizes selected published cases of WE worldwide. Beginning with Wernicke’s original description of *polioencephalitis haemorrhagica superior* [3], symmetrical periventricular and brainstem lesions were found in the postmortem examination of three patients presenting acute confusional state, ocular abnormalities, and gait instability. Although two of the patients were chronic alcohol users, the syndrome was not exclusive to alcoholism, as Wernicke also described a malnourished nonalcoholic woman [11]. Nutritional deficiency is considered the primary pathogenic factor underlying these neurological changes, and numerous subsequent reports have supported this observation.

**TABLE 2:** Differential diagnosis and exclusions.

Differential diagnosis	Reason for consideration	Reason for exclusion in this case
Wernicke’s encephalopathy	Altered mental status, prolonged vomiting, poor oral intake, nutritional deficiency, characteristic bilateral symmetrical MRI findings	Confirmed by fulfillment of the Caine criteria (dietary deficiency and altered mental status), characteristic MRI findings, and rapid clinical improvement following high‐dose intravenous thiamine.
Acute necrotizing encephalopathy	Bilateral thalamic involvement on MRI	No preceding viral illness, fever, seizures, or rapid fulminant encephalopathy. MRI lacked hemorrhagic or necrotic changes typical of acute necrotizing encephalopathy.
Viral encephalitis	Altered mental status	No clinical features suggestive of central nervous system infection (e.g., fever, seizures, meningeal signs), and MRI findings were more typical of WE than encephalitis.
Bilateral thalamic infarction (Artery of Percheron infarction)	Bilateral thalamic lesions and altered consciousness	Clinical presentation was subacute rather than abrupt, there were no focal neurological deficits, and MRI findings were symmetrical and characteristic of WE rather than vascular infarction.
Deep cerebral venous thrombosis	Bilateral thalamic abnormalities may occur	No severe headache, seizures, papilledema, or radiological evidence of venous thrombosis. Clinical course and response to thiamine favored WE.
Osmotic demyelination syndrome	Altered mental status with electrolyte disturbances	No history of rapid correction of hyponatremia, and MRI distribution was not typical of pontine or extrapontine myelinolysis.
Other metabolic or toxic encephalopathies	Altered sensorium and electrolyte abnormalities	Electrolyte abnormalities alone did not explain the characteristic MRI findings. Rapid neurological recovery after thiamine replacement strongly supported WE.

In the modern literature, hyperemesis gravidarum has been a major precipitating factor for malnutrition and subsequent neurological manifestations. In most reports, there was a combination of altered mental state, such as confusion and cognitive slowing, with ocular symptoms such as nystagmus, ophthalmoplegia, and blurred vision [6, 12–14]. These features closely resemble Wernicke’s original observation [3]. Several atypical symptoms were also reported, often causing diagnostic dilemmas such as Choubane et al. reported peripheral neurological symptoms before developing encephalopathy [12]. Khallati et al. described psychiatric manifestation such as delusional thinking and visual and auditory hallucination [14], and Szablewoska et al. found memory lapses and unawareness regarding own pregnancy too [15]. Bensaid et al. reported features resembling preeclampsia [13], Alaithan et al. described right third and sixth cranial nerve palsies [5], and Risq et al. found prominent vision loss close to blindness in both eyes [16].

The radiological findings are almost similar in all the studies which is bilateral symmetrical hyperintensities shown in the medial thalami, mammillary bodies, and periaqueductal gray matter on T2‐weighted and FLAIR MRI [6, 13–19]. These findings closely mirror the postmortem autopsy findings of Wernicke’s in the first described book [3].

Despite the diagnostic challenges, nearly all reported cases demonstrated clinical improvement following intravenous thiamine administration, with some reports documenting complete resolution of both neurological symptoms and radiological abnormalities [6]. Overall, the reviewed literature (Table 3) demonstrates an uncommon neurological complication developed in pregnancy with persistent vomiting, often causes confusion, ataxia, or ocular abnormalities leading to many grave situations such as visual loss [19]. In such cases, early consideration of thiamine deficiency, even in the absence of alcohol use or characteristic imaging findings, may help prevent significant neurological morbidity [6, 13, 15–17, 19].

**TABLE 3:** Literature review in concise.

Author year references	Case number/methodology	Age/sex	Clinical presentation	Radiology/MRI findings	Any special comment
Wernicke (1881) [3]	Number of cases: 3 Methodology: Clinical and bed side observation with postmortem neuropathological examination (autopsy)		The patients described here presented with acute mental confusion and clouding of consciousness, accompanied by reduced responsiveness. Neurological manifestation like inability to walk and ocular abnormalities such as ophthalmoplegia and gaze palsy were also present.	Postmortem autopsy finding: Bilateral symmetrical changes are seen in periventricular and brainstem regions, more prominent in third and fourth ventricles and mammillary bodies. There were punctate hemorrhages which were consistent with an acute inflammatory–hemorrhagic process. Mostly periaqueductal gray matter and diencephalic structures were involved sparing cerebral cortex.	Wernicke named the condition “*Polioencephalitis haemorrhagica superior*” in the book. This is the first time this encephalopathy is reported.

Szablewska et al. (2025) [15]	Sample size: 1 (case report)	21‐year‐old, female	A 15‐week primigravida presented with persistent vomiting and weight loss, followed by a syncopal episode associated with cognitive slowing, memory impairment, disorientation, transient unawareness of her pregnancy.	MRI of brain findings: Symmetrical bilateral medial thalamic hyperintensity on T2‐weighted and TIRM‐T2 imaging, with no other detected abnormalities	By treating with intravenous thiamine, neurological symptoms improved rapidly.

Nabila (2025) [12]	Sample size: 1 (case report)	28‐year‐old, female	A Primigravida with 16 weeks of gestation presented with persistent vomiting, weight loss, and progressive muscle weakness, followed by acute confusion (GCS 13) and bilateral multidirectional nystagmus.	MRI of brain findings: Symmetrical T2/FLAIR hyperintensities in the thalami, mammillary bodies, and periaqueductal areas, consistent with findings of WE.	This case highlights an atypical presentation of WE (WE), initially misleading due to its peripheral neurological symptoms.

Bensaid et al. (2025) [13]	Sample size: 1 (case report)	38‐year‐old, female	A 27‐week primigravida presented with severe epigastric pain and intractable vomiting for over a month, followed by an acute confusional state (GCS 13/15), nystagmus, cerebellar ataxia, and paresthesia of both the upper and lower limbs, with concomitant 3+ proteinuria.	MRI of brain findings: Bilateral, symmetrical signal abnormalities in the dorsomedial thalami, periaqueductal regions, and mammillary bodies. Contrast CT scan revealed no abnormality.	The presence of preeclampsia with WE delayed its diagnosis process and complicated the case.

Khallati et al. (2025). [14]	Sample size: 1 (case report)	21‐year‐old, female	A primigravida at 16 weeks of gestation presented with persistent vomiting and generalized weakness, followed by psychomotor slowing and sudden vision loss associated with neuropsychiatric symptoms including visual and auditory hallucinations and delusional thoughts.	MRI of brain findings: Bilateral symmetrical periaqueductal T2/FLAIR hyperintensities consistent with WE.	

Borgemenke et al. (2023). [17]	Sample size: 1 (case report)	22‐year‐old, female	A 13‐week primigravida presented with altered mental status, blurred vision, and dizziness, manifesting as disorientation and a trance‐like state with prolonged staring at ceiling.	A noncontrast CT scan of head CT was unremarkable and showed no evidence of mass lesion, acute hemorrhage, and infarct. MRI brain showed abnormal symmetric hyperintensities within the bilateral thalami.	

Alaithan et al. (2023) [5]	Sample size: 1 (case report)	23‐year‐old, female	A primigravida at 17 weeks of gestation presented with persistent vomiting for 3 months, accompanied by dizziness, lower limb weakness, and auditory and visual hallucinations, progressing to altered consciousness (GCS 13/15), and right third and sixth cranial nerve palsies.	MRI brain showed mesial temporal lobes atrophic changes and no acute intracranial pathology.	

Current Case	Sample size: 1 (case report)	40‐year‐old, female	Patient presented with history of missed abortion at 17 weeks of gestation, managed with dilatation and curettage, presented with persistent vomiting, poor oral intake, and generalized weakness, followed by confusion, disorientation, impaired attention, and altered sensorium (GCS‐ 11/15), without ocular abnormalities, ataxia, or focal neurological deficits.	MRI brain shows symmetrical T2/FLAIR hyperintensity in both medical thalami, periaqueductal gray matter and mammillary bodies, no abnormal enhancement or diffusion restriction.	

In conclusion, this case reinforces the need to consider WE in nonalcoholic patients presenting with malnutrition, prolonged anorexia, or metabolic imbalance, particularly following obstetric events. Increased awareness and early empirical thiamine administration are essential to improve outcomes and reduce long‐term neurological sequelae.

### 3.1. Limitations

Weight loss and ketonuria were not documented at the time of admission and, therefore, cannot be reported.

## Author Contributions

Himel Kumar Biswas contributed to a major role in the conceptualization of the study, diagnosis, acquisition of data, study design, data analysis, drafting of the manuscript, and approval of the final manuscript.

Monisha Basak contributed to patient management, investigations, visualization, validation writing original draft, and revising the manuscript.

Mosarrat Mahjabeen contributed to revising the manuscript for content, including literature review writing, and played a major role in data acquisition as well as analysis and interpretation of data.

Nazia Ahmed contributed in drafting the original manuscript, investigations, and acquisition of data.

Rama Biswas contributed to diagnosis, patient management, visualization, validation, revising the manuscript, and supervision.

## Funding

This study received no financing from any governmental, corporate, or nonprofit organization.

## Disclosure

All authors read and approved the final draft.

## Ethics Statement

The patient was treated in accordance with the Declaration of Helsinki. According to the Square Hospital Ltd. ethical board, no approval is needed for the publication of case reports and case series.

## Consent

The authors have nothing to report.

## Conflicts of Interest

The authors declare no conflicts of interest.

## Data Availability

The data that support the findings of this study are available from the corresponding author upon reasonable request.
